# p-Hydroxybenzyl Alcohol Antagonized the ROS-Dependent JNK/Jun/Caspase-3 Pathway to Produce Neuroprotection in a Cellular Model of Parkinson’s Disease

**DOI:** 10.3390/nu14235002

**Published:** 2022-11-24

**Authors:** Mei-Chou Lai, Wayne-Young Liu, Shorong-Shii Liou, I-Min Liu

**Affiliations:** 1 Department of Pharmacy and Master Program, Collage of Pharmacy and Health Care, Tajen University, Pingtung County 90741, Taiwan; 2 Department of Urology, Jen-Ai Hospital, Taichung 41265, Taiwan; 3 Center for Basic Medical Science, Collage of Health Science, Central Taiwan University of Science and Technology, Taichung City 406053, Taiwan

**Keywords:** Parkinson’s disease, p-hydroxybenzyl alcohol, neurodegeneration, 6-OHDA, SH-SY5Y cells

## Abstract

Parkinson’s disease (PD) is a progressive disorder that affects brain nerve cells responsible for body motion and remains incurable. p-Hydroxybenzyl alcohol (HBA) is the primary phenolic compound in Gastrodiae Rhizoma, known for its therapeutic benefits against neurodegeneration. However, the protective effect of HBA against Parkinson’s disease (PD) remains unclear. The objective of this study was to evaluate the neuroprotective effects of HBA in vitro 6-hydroxydopamine (6-OHDA)-induced PD model in SH-SY5Y cells. SH-SY5Y cells were pretreated with various concentrations of HBA for 1 h and incubated with 100 μmol/L 6-OHDA for 24 h to induce cellular lesions. 2,5-Diphenyl-2H-tetrazolium bromide was used to detect cellular viability. 2′,7′-dichlorofluorescin oxidation detects reactive oxygen species (ROS). The enzyme-linked immunosorbent assay was used to determine the activities of superoxide dismutase, catalase, and glutathione peroxidase. The cellular mitochondrial function was identified through the collapse of the mitochondrial membrane potential, the release of cytochrome c, and the synthesis of mitochondrial ATP. Expression of pro-and anti-apoptotic factors was measured by Western blot. HBA enhanced cell viability, blocked ROS overproduction, and reduced antioxidant activities induced by 6-OHDA. HBA also reduced mitochondrial dysfunction and cell death caused by 6-OHDA. Moreover, HBA reversed the 6-OHDA-mediated activation of c-Jun N-terminal kinase, the downregulation of the Bcl-2/Bax ratio, the Apaf-1 upregulation and the induction of caspase-9, caspase-3, and PARP cleavage. This study shows that the protective effects of HBA against 6-OHDA-induced cell injury provide the potential preventive effects of HBA, making it a promising preventive agent for PD.

## 1. Introduction

Parkinson’s disease (PD) is the second most widespread neurodegenerative disease following Alzheimer’s disease (AD) [1]. In addition, as the average age of the population continues to rise, the number of PD patients is expected to rise in the elderly [1]. The pathogenic feature of PD is the progressive and devastating neurodegeneration of dopaminergic neurons in substantia nigra pars compacta, which limits the facility and balance of motion [1]. The primary motor symptoms of PD are slowness of movement, resting tremors, stiffness, and postural instability [1]. Although current management is aimed at providing symptomatic relief and slowing the progress of PD, there are no effective treatments for this disease because of the complex mechanisms responsible for the neural degeneration of PD that remain entirely elusive [2].

Accumulating evidence has been proven that a major contributor to the loss of dopaminergic neurons in the brain of PD is oxidative stress once the production of reactive oxygen species (ROS) overwhelms the intrinsic anti-oxidant defenses [3]. Mitochondria are regarded as the primary source of ROS within the cell [4]. Cellular ROS accumulation can activate multiple stress-sensitive serine kinase cascades; one such is C-Jun-N-terminal kinase (JNK), belonging to the serine/threonine protein kinase superfamily of mitogen-activated protein kinase (MAPK) [5]. Activated JNK is known to induce the dissipation of mitochondrial membrane potential (MMP) and cytochrome c release required for activating downstream caspases; in the same way, the activation of transcription factors such as AP-1 or c-Jun ultimately leads to apoptosis, thus regulating cellular processes involving the progression of PD [6]. Therapeutic strategies for reducing abnormalities, such as neural oxidative stress and mitochondrial dysfunction, as mentioned above, might be candidates for treatment of PD [7]. Furthermore, because of the critical role of JNK in the pathogenesis of PD, the blocked JNK pathway provides a molecular target for PD treatment [8].

p-Hydroxybenzyl alcohol (HBA), also called gastrodigenin, is the primary phenolic compound from Gastrodiae Rhizoma, known as Tianma in Chinese [9]. Gastrodiae Rhizoma is the dried rhizome of *Gastrodia elata* Blume (Orchidaceae family), which is medicine and food homology that can be scientifically and practically used as both food and medicine [10]. Gastrodiae Rhizoma has been used for centuries in traditional Chinese medicine to treat several disorders linked to the central nervous system, such as cerebral ischemic lesions, epilepsy, and amnesia, and is listed in the Chinese Pharmacopoeia [11]. HBA and its glucoside, gastrodin, were regarded as quality control markers for *G. elata* [9]. Gastrodin has been shown to enter the blood–brain barrier (BBB) and is subsequently biotransformed into HBA following entrance into the central nervous system [12]. HBA, along with gastrodin, was the major active ingredient in Gastrodiae Rhizoma in the treatment of various neurological diseases [12]. Past results indicate that HBA with neuroprotective effects on the post-ischemic brain due to this compound may against zinc induces toxicity in neurons and astrocytes, the cellular model of vascular-type dementia [13]. Daily oral administration of HBA (40 mg/kg) to a mouse for 28 days has been documented as potentially improving scopolamine-induced learning and memory deficits [14]. HBA has neuroprotective effects against AD by reducing proinflammatory mediators to inhibit cell death in an AD cell model of BV-2 microglial cells treated with amyloid ß [15]. HBA also prolongs the life of *Caenorhabditis elegans* through documented worm models of age-associated neurodegenerative diseases [16]. Although HBA appears to have a relatively higher activity against neurodegenerative disease as a result of its antioxidant and free radical scavenging activity [17], the protective effect of HBA against PD is not entirely apparent, and cellular mechanisms must be highly characterized.

PD models induced by neurotoxins are widely used to understand the mechanisms of neural degeneration in PD [18]. 6-Hydroxydopamine (6-OHDA) is a selective catecholaminergic nerve toxin that may take up to catecholaminergic terminals to generate ROS, causing mitochondrial dysfunction and leading to cell death [19]. Thus, 6-OHDA has been commonly used to create disease models of PD in vitro and in vivo [19]. Current PD research is carried out primarily with permanently established neuronal cell models, particularly because of their human origin with catecholaminergic nerve properties and maintenance easily [20]. SH-SY5Y cells treated by 6-OHDA are widely used as a valuable cellular death model of dopaminergic neuronal cells to mimic pathophysiological degeneration of PD. [21]. Therefore, this study aimed to elucidate the potential effects and protective mechanisms of HBA against PD in a cell-based model of PD built by SH-SY5Y cells treated with 6-OHDA.

## 2. Materials and Methods

### 2.1. Cell Culture

The SH-SY5Y cells (no. CRL-266) bought from American Type Culture Collection (Manassas, VA, USA) were grown with DMEM complemented by 10% fetal bovine serum and 1% streptomycin/penicillin, in conditions with a humidified atmosphere of 5% CO_2_ and 95% air at 37 °C. Following adherence, cells were differentiated with 10 μmol/L retinoic acid (Sigma-Aldrich, St. Louis, MO, USA). All experiments were treated after the five-day differentiation period.

### 2.2. Cell Model

Cells were seeded in 6-well plates at a density of 2 × 10^6^ cells per well, and cultures were passaged by dissociation in 0.05% (*w*/*v*) trypsin in phosphate-buffered saline (PBS) pH 7.4 upon the confluence. For the pretreatment studies, the cells were treated with HBA (IUPAC name: 4-(Hydroxymethyl)phenol; Sigma Chemical Co., St. Louis, MO, USA; Cat. # H20806, purity ≥ 99%) at indicated concentrations (40, 80, 120, 160 µmol/L), 1 mmol/L N-acetyl-L-cysteine (NALC; Sigma Chemical Co., St. Louis, MO, USA; Cat. # A9165) or 10 µmol/L SP600125 (Sigma Chemical Co., St. Louis, MO, USA; Cat. # 420119) for 1 h, followed by exposure to a range of concentrations (25, 50, 100, 150, and 200 µmol/L) of 6-OHDA (IUPAC name:5-(2-aminoethyl)benzene-1,2,4-triol; Sigma-Aldrich, St. Louis, MO, USA; Cat. # H4381) for 24 h with no medium change. The powders of the experimental compounds were dissolved in dimethyl sulfoxide (DMSO, Sigma-Aldrich, St. Louis, MO, USA; Cat. # D8418) to form a 1 mmol/L stock solution and then diluted in a culture medium at concentrations appropriate for later experiments. The final concentration of DMSO was below 0.1% (*v*/*v*), which is not usually an observable toxic effect for most cells [22]. Each condition was tested with three cell wells for each replicate, and each experiment was carried out a minimum of five separate times

### 2.3. Cell Viability Assay

The 3-(4,5-dimethyl-2-yl)-2,5-diphenyl tetrazole bromide (MTT) assay carried out by the MTT cell proliferation assay kit (cat # ab211091, Abcam plc., Cambridge, MA, USA) was used to determine cell viability [23]. MTT is converted to water-insoluble precipitate within the mitochondria of cells and shows violet. The solubilizing solvent was used to dissolve the insoluble violet product, and the colored solution was measured by a microplate reader (SpectraMax M5, Molecular Devices, Sunnyvale, CA, USA) at 570 nm. Cellular viabilities were expressed as a percentage of the vehicle-treated control.

### 2.4. Detection for Intracellular ROS Levels

The level of intracellular ROS was detected with dichloro-dihydro-fluorescein diacetate (DCFH-DA), as directed by the manufacturer (Sigma-Aldrich, St. Louis, MO, USA) [24]. In brief, DCFH-DA was diluted with DMEM at 10 μmol/L, then added to the cells for an incubation period of 20 min at 37 °C. Once treated with DCFH-DA, the cells were washed three times with PBS. The images were taken using a phase contrast fluorescence microscope (Nikon, model 80i). The DCF fluorescence was recorded from a multifunctional microplate reader (SpectraMax M5, Molecular Devices, Sunnyvale, CA, USA) with an excitation and emission wavelength of 488 nm and 525 nm, respectively. The change in ROS levels was expressed as a percentage compared to the control.

### 2.5. Determination of the Activities of Antioxidants

The enzyme-linked immunosorbent assay was used for the detection of superoxide dismutase (SOD; EC 1.15.1.1), glutathione peroxidase (GSH-Px; EC 1.11.1.9), and catalase (CAT; EC 1.11.1.6). The commercial activity colorimetric assay kits of SOD (Cat. # K335), GPx (Cat. # K762), and CAT (Cat. # K773) were purchased from Bio Vision, Inc. (San Francisco, CA, USA). SOD, GPx, and CAT activities were calculated to measure absorption at 450 nm, 340 nm, and 570 nm, respectively, using a microplate reader (SpectraMax M5, Molecular Devices, Sunnyvale, CA, USA) and expressed as units per milligram protein. Protein concentration was determined using the Bradford dye binding method.

### 2.6. Measurement of Mitochondrial Membrane Potential (MMP)

A commercial kit containing 5,5′,6,6′-tetrachloro-1,1′,3,3′-tetraethylbenzimi-dazolylcarbocyanin iodine (JC-1) (Abcam plc., Cambridge, MA, USA) was used to measure MMP. Cells seed on a 96-well plate with a density of 1 × 10^4^ cells/well and incubated with growth medium of 20 µmol/L JC-1 at 37 °C for 30 min. Subsequently, the cells were centrifuged at 2500 rpm for 5 min, and the pellets were resuspended in 0.5 mL of PBS. The emission of the green aggregate monomer form (530 nm) and the red aggregate form (590 nm) was determined with a fluorescence spectrophotometer (SpectraMax M5, Molecular Devices, Sunnyvale, CA, USA). The red/green fluorescent intensity ratio was calculated to determine the changes in the MMP [25].

### 2.7. Measurement of ADP and ATP Levels 

The ADP/ATP Ratio Assay Kit (Cat. # ELDT-100) from BioAssay Systems (Hayward, CA, USA) relies on the ability of luciferase to generate light in the presence of its luciferin substrate [26]. In summary, upon completion of treatment, cells were lysed with 10% tricholoroacetic acid, neutralized with 1 mol/L of KOH, and diluted with 100 mmol/L HEPES buffer (pH 7.4). The first phase of the trial involved the luciferase-catalyzed reaction of cellular ATP and D-luciferin, which produced a luminescent signal. Subsequently, ADP was converted to ATP by enzymatic reaction, and the newly formed ATP reacted with D-luciferin. The second light intensity was the total amount of ADP and ATP. The calculated ADP/ATP ratio was standardized against the total protein content in the samples.

### 2.8. Measurement of Cytochrome C Release 

After treatment, the cells were homogenized, and the lysate spun twice at 800× *g* for 20 min. The resulting supernatant was centrifuged at 10,000× *g* for 15 min to produce the mitochondrial pellet. The rest of the supernatant was centrifuged at 16,000× *g* for 25 min to obtain a cytosolic fraction. Following isolation of the mitochondria and cytosolic fraction, the cytochrome C ELISA kit (Abcam plc., Cambridge, MA, USA; Cat. # ab110172) was used to measure the level of cytochrome C according to the manufacturer’s instructions. Cytochrome c is immunocaptured within the wells determined by adding a cytochrome c-specific antibody conjugated with horseradish peroxidase. This peroxidase converts the substrate of colorless to blue, which was stopped by adding 100 μL of 1.5 N HCl to each well and then measured at 450 nm. Protein levels were measured using a Bio-Rad protein analysis.

### 2.9. Study of Apoptotic DNA Fragmentation 

The cell death detection ELISA kit (Roche Molecular Biochemicals, Mannheim, Germany; Cat. # 11774425001) was used to quantitatively detect DNA fragments associated with cytoplasmic histones as a result of induced cell death. At the end of the treatment, cytoplasmic extracts from cells were used as an antigen source in a sandwich ELISA with a primary anti-histone mouse monoclonal antibody-coated to the microtiter plate and a second anti-DNA mouse monoclonal antibody coupled to peroxidase. The amount of peroxidase retained in the immune complex was determined photometrically by incubation with 2,2′-azino-di-(3-ethylbenzthiazoline sulfonate) (ABTS) as a substrate for 10 min at 20 °C. A microplate reader (SpectraMax M5, Molecular Devices, Sunnyvale, CA, USA) measured the color change at a wavelength of 405 nm. The optical density reading at 405 nm (OD405) was standardized to milligrams of protein used in the test and declared an apoptotic DNA fragmentation index.

### 2.10. Measurement for Caspases and Poly (ADP-Ribose) Polymerase (PARP) Activities

The caspases-9 activity was determined using a caspase colorimetric test kit (Abcam plc., Cambridge, MA, USA; Cat. # ab65608) based on the spectrophotometric detection of the chromophore p-nitroanilide (pNA) after caspase separation of the substrate labelled acetyl-Leu-Glu-His-Asp-pNA. Caspase-3 colorimetric activity assay kit (Abcam plc., Cambridge, MA, USA; Cat. # ab39401) was used to measure caspase-3 activity according to the spectrophotometric detection of pDNA following the cleavage of the labeled substrate Asp-Glu-Val-Asp-pNA. The free pNA was quantified using a microplate reader (SpectraMax M5, Molecular Devices, Sunnyvale, CA, USA) at 405 nm.

PARP activity levels have been measured by PARP/Apoptosis colorimetric Assay Kit (R&D Systems, Minneapolis, MN, USA; Cat. # 4684-096-K) as per the manufacturer’s protocol. The activity of PARP-1 was assessed by semi-quantitative measurement of the amount of poly (ADP-ribose) deposited on immobilized histone proteins and detection of absorbance values at the 450 nm wavelength using a microplate reader (SpectraMax M5, Molecular Devices, Sunnyvale, CA, USA). All the values were compared with those obtained from the vehicle-treated control.

### 2.11. Western Blot Analysis

Cells were collected and lysed in an iced radioimmunoprecipitation buffer for 30 min. Protein concentration was determined by the Bradford method, and 50 μg of protein in each sample was used for Western blot analysis. Protein was separated on 10% sodium dodecyl sulfate-polyacrylamide gel and transferred electrophoretically to polyvinylidene difluoride membranes.

The membranes were sealed with 5% non-fat dry milk in tris-buffered saline with Tween for 3 h at room temperature, then incubating at night at 4 °C with primary antibodies against Apaf-1(Cat. # 5088), cleaved caspases-9 (Asp353) (Cat. # 9509), cleaved caspases-3 (Asp175) (Cat. # 9661), cleaved caspases-PARP (Asp214) (Cat. # 9544), JNK (Cat. # 9252), p-JNK (Thr 183/Tyr 185) (Cat. # 9251), c-Jun (Cat. # 9162), p-c-Jun (Ser73) (Cat. # 9164), Bcl-2 (Cat. # 2876), Bax (Cat. # 2772), or β-actin (Cat. # 4967). All of the antibodies were purchased from Cell Signaling Technology, Inc. (Danvers, MA, USA) and used in dilutions of 1:1000. After washing with tris-buffered saline with 0.1% Tween^®^ 20 detergent, blots were incubated with the secondary antibodies at room temperature for 1.5 h before visualization with chemoluminescence (Amersham Biosciences, Amhershem, UK). The densities of the bands were quantified by means of the densitometric analysis using the densitograph software ATTO and expressed in connection with the β-actin. All values were normalized by adjusting the density of untreated control samples to 1.0, and the expression differences represent a “fold change.” Cells were collected from five separate experiments.

### 2.12. Statistical Analysis

Data are expressed as the mean ± standard deviation (SD). The statistical analysis was performed in Systat SigmaPlot version 14.0 (Systat Software Inc., San Jose, CA, USA). If no special description was available, significant differences from the vehicle controls were assessed through one-way ANOVA analysis followed by the Dunnett’s test as post hoc test. The differences were statistically significant at *p* < 0.05.

## 3. Results 

### 3.1. HBA Rescues SH-SY5Y Cells from 6-OHDA-Induced Neurotoxicity

**Figure 1:**
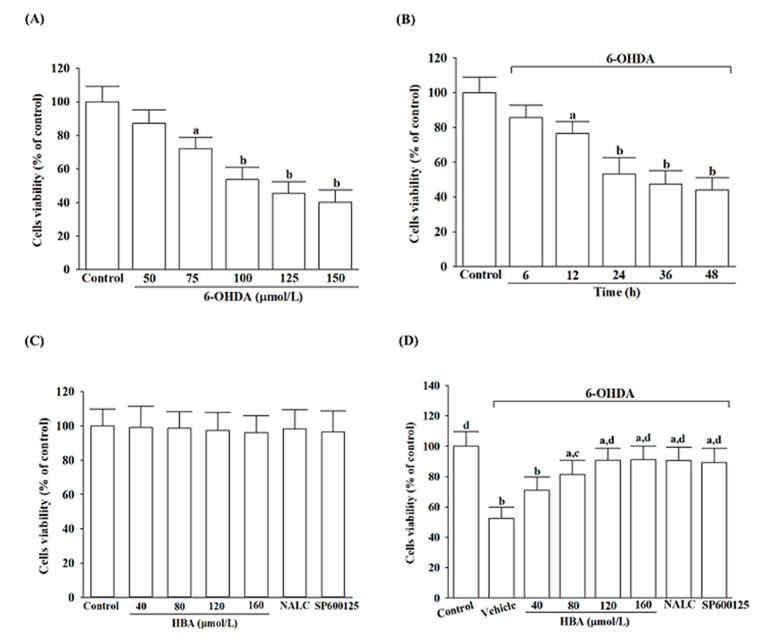
HBA rescues SH-SY5Y cells from the neurotoxicity induced by 6-OHDA. (**A**) Cell viability was assessed for various concentrations of 6-OHDA that were incubated with SH-SY5Y cells over a 24 h period. (**B**) Cell viability was evaluated for SH-SY5Y cells incubated with 100 μmol/L 6-OHDA for a period. (**C**) Cell viability was evaluated when SH-SY5Y cells were incubated with various concentrations of HBA, 1 mmol/L NALC, or 10 µmol/L SP600125 for 24 h without exposure to 100 μmol/L 6-OHDA. (**D**) Cell viability was evaluated when SH-SY5Y cells were pretreated with different concentrations of HBA, 1 mmol/L NALC, or 10 µmol/L SP600125 for 1 h and then exposed to 100 μmol/L of 6-OHDA for another 24 h. Cell viability was determined with an MTT assay and expressed as a percentage of untreated cells, considering the control group. The results are shown as the mean ± SD of five independent experiments (*n* = 5) performed in triplicate. ^a^ *p* < 0.05 and ^b^ *p* < 0.01 compared to the data from untreated control group (control); ^c^ *p* < 0.05 and ^d^ *p* < 0.01 compared to the data from cells exposed to 6-OHDA with no treatment.

Cell viability was determined after incubation with 6-OHDA in the concentration correlation experiment (50–150 μmol/L) and at 6–48 h in the time experiment. A significant decrease in cell viability after 24 h incubating of SH-SY5Y cells with an increasing concentration of 6-OHDA (Figure 1A). 6-OHDA (100 μmol/L) decreases cellular viability in a time-dependent trend. (Figure 1B). The concentration of 6-OHDA, which resulted in a 50% cell inhibition at 24 h, was 100 μmol/L compared with the vehicle-treated control group (Figure 1B). Based on the above results, SH-SY5Y cells were exposed to 100 μmol/L 6-OHDA for 24 h to induce neural damage in subsequent experiments.

The cell viability of SH-SY5Y cells was not affected by HBA (40–160 µmol/L) treatment alone (Figure 1C). Cell death was not caused by NALC (1 mmol/L) or SP600125 (10 µmol/L) on its own (Figure 1C). HBA prevented cell death caused by 6-OHDA in a concentration-dependent manner (Figure 1D). There was no significant difference in the elevation of 6-OHDA-induced lower cell viability between HBA at a concentration of 120 µmol/L (90.6%) and 160 µmol/L (91.2%) (Figure 1D). The potential concentration of HBA to enhance cell survival was considered at 120 µmol/L, which was selected for further studies to clarify the protective effects of HBA on 6-OHDA-induced neurotoxicity in SH-SY5Y cells. Cell viability is maintained at 90.5% and 89.3% in 6-OHDA-stimulated cells pretreated for 1 h with 1 mmol/L of NALC and 10 µmol/L of SP600125, respectively (Figure 1D).

### 3.2. HBA Reduces SH-SY5Y Cells from 6-OHDA-Induced Oxidative Stress

**Figure 2:**
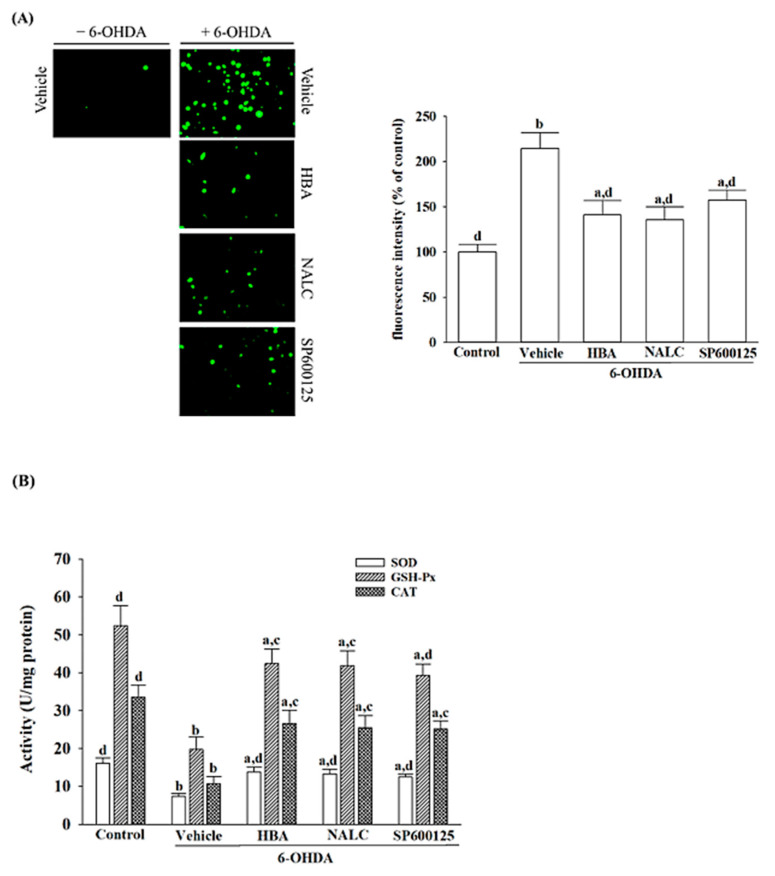
HBA reduces SH-SY5Y from the oxidative stress induced by 6-OHDA. SH-SY5Y cells were pretreated with HBA (120 µmol/L), NALC (1 mmol/L), or SP600125 (10 µmol/L) for 1 h and then exposed to 6-OHDA (100 μmol/L) for another 24 h. (**A**) Fluorescence images representative of intracellular ROS production were detected using DCFH-DA fluorescent probes (200× *g* magnification). The ROS fluorescence intensity is represented as a percentage in comparison with untreated control cells. (**B**) The activities of SOD, GSH-Px, and CAT were identified using commercial analysis packages. The results are shown as the mean ± SD of five independent experiments (*n* = 5) performed in triplicate. ^a^ *p* < 0.05 and ^b^ *p* < 0.01 compared to the data from untreated control group (control); ^c^ *p* < 0.05 and ^d^ *p* < 0.01 compared to the data from cells exposed to 6-OHDA with no treatment.

6-OHDA raised the level of intracellular ROS in SH-SY5Y cells to 2.2-fold of non-treated vehicle controls (Figure 2A). Pretreatment of HBA (120 μmol/L) reduced 6-OHDA-induced ROS production in SH-SY5Y cells by 37.2%; the results were similar to those obtained with 1 mmol/L of NALC (39.6% reduction) or 10 µmol/L of SP600125 (30.8% reduction; Figure 2A).

6-OHDA may decrease not only SOD activity but also GSH-Px and CAT activity in SH-SY5Y cells (Figure 2B). In SH-SY5Y cells pretreated with 120 μmol/L HBA, it significantly increased intracellular activity in SOD, GSH-Px, and CAT to 3.1-, 3.2-, and 2.7-fold compared with 6-OHDA alone-treated cells, respectively (Figure 2B). The lower intracellular activities in SOD, GSH-Px, and CAT caused by 6-OHDA were elevated to 3.0-, 3.1-, and 2.5-fold when SH-SY5Y cells received 1 mmol/L NALC pretreatment (Figure 2B). The pretreatment cell with SP600125 (10 μmol/L) performed intracellular activities in SOD, GSH-Px, and CAT cells at 3.1-, 3.2-, and 2.7-fold those of the 6-OHDA-treated cells, respectively (Figure 2B).

### 3.3. HBA Reduces SH-SY5Y Cells of 6-OHDA-Induced Mitochondrial Dysfunction

**Figure 3:**
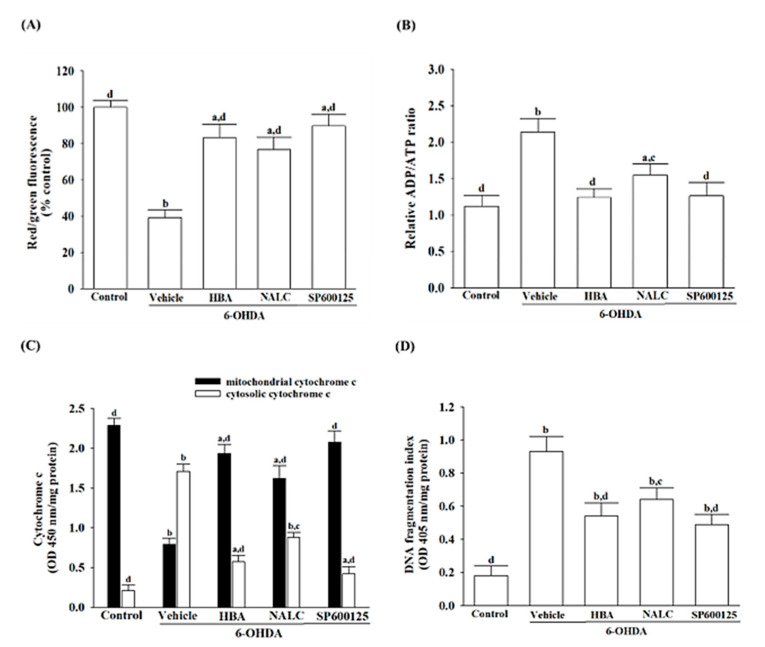
HBA reduces SH-SY5Y cells of 6-OHDA-induced mitochondrial dysfunction. SH-SY5Y cells were pretreated with HBA (120 µmol/L), NALC (1 mmol/L), or SP600125 (10 µmol/L) for 1 h and then exposed to 6-OHDA (100 μmol/L) for another 24 h. (**A**) The potential of the mitochondrial membrane was measured using the JC-1 fluorescence probe. (**B**) The ADP/ATP ratio in cells was measured using a commercial assay kit based on the bioluminescent detection of ADP and ATP levels. (**C**) Cytochrome c concentrations were determined by immunoassay for mitochondrial and cytosolic fractions. (**D**) The extent of apoptotic DNA fragmentation was quantified by using the cell death ELISA kit. The results are shown as the mean ± SD of five independent experiments (*n* = 5) performed in triplicate. ^a^ *p* < 0.05 and ^b^ *p* < 0.01 compared to the data from untreated control group (control); ^c^ *p* < 0.05 and ^d^ *p* < 0.01 compared to the data from cells exposed to 6-OHDA with no treatment.

MMP in 6-OHDA-treated SH-SY5Y cells was reduced to 39.2% of controls for untreated vehicles (Figure 3A). Pretreatment of SH-SY5Y cells with HBA (120 µmol/L) reduced MMP disturbance induced by 6-OHDA (Figure 3A). The reduction of MMP was also alleviated in SH-SY5Y due to 6-OHDA in the pre-treated NALC (1 mmol/L) or SP600125 (10 μmol/L) group (Figure 3A).

The 2.1-fold ADP/ATP ratio elevation of controls for untreated vehicles was obtained in SH-SY5Y cells stimulated with 6-OHDA (Figure 3B). The higher ADP/ATP ratio in 6-OHDA-treated SH-SY5Y cells was reduced by HBA (120 μmol/L) pretreatment with a 42.1% reduction (Figure 3B). A significant increase in the ADP/ATP ratio caused by 6-OHDA was not observed in the pre-treated NALC (1 mmol/L) or SP600125 (10 μmol/L) group: less than 32.7% and 41.1% of the values for 6-OHDA, respectively (Figure 3B).

Decreased mitochondrial cytochrome c levels, but in parallel, increased cytosolic cytochrome c levels were observed in SH-SY5Y cells receiving 6-OHDA stimulation (Figure 3C). Pretreatment of SH-SY5Y cells with HBA inhibited the 6-OHDA-induced cytochrome c release from mitochondria to the cytoplasm (Figure 3C). NALC (1 mmol/L) or SP600125 (10 μmol/L) pretreatment also prevented SH-SY5Y cells from releasing 6-OHDA-induced cytochrome c from mitochondria into the cytoplasm (Figure 3C).

6-OHDA increased apoptotic DNA fragmentation in SH-SY5Y cells to 5.1-fold of the untreated control (Figure 3D). In pretreatment with HBA (120 μmol/L), NALC (1 mmol/L), or SP600125 (10 μmol/L), apoptotic DNA fragmentation decreased by 41.9, 31.1, and 47.3% in SH-SY5Y cells grown in 6-OHDA, respectively (Figure 3D).

### 3.4. HBA Reduces Mitochondrial Apaf-1/Caspase-9 Pathway-Mediated Caspases Activation Induced by 6-OHDA

**Figure 4:**
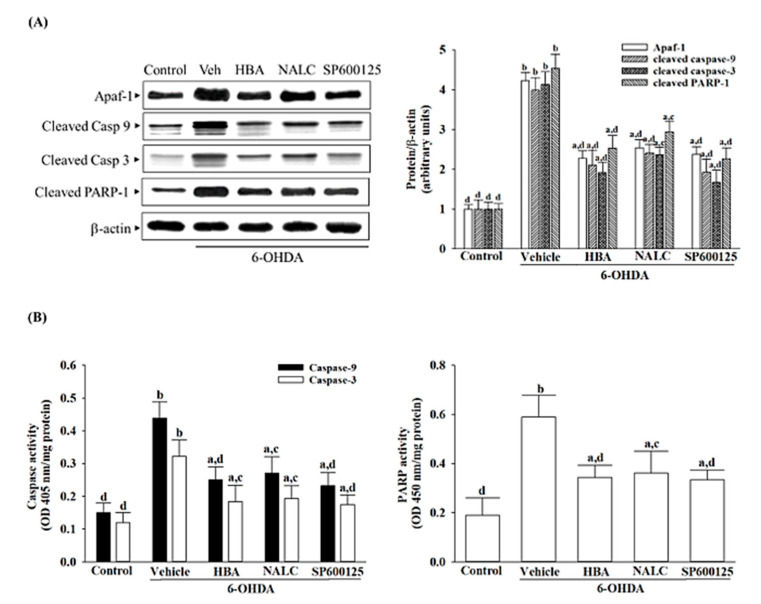
HBA reduces mitochondrial Apaf-1/caspase-9 pathway-mediated caspase-3 activation induced by 6-OHDA. SH-SY5Y cells were pretreated with HBA (120 µmol/L), NALC (1 mmol/L), or SP600125 (10 µmol/L) for 1 h and then exposed to 6-OHDA (100 μmol/L) for another 24 h. (**A**) Western blot was carried out to detect the expression of Apaf-1, cleaved caspase-9 (Casp 9), cleaved caspase-3 (Casp 3), and cleaved PARP-1. Band densities of proteins have been normalized to β-actin. (**B**) Caspase-9, caspase-3, and PARP activities were evaluated using the commercial colorimetric assay kits. The results are shown as the mean ± SD of five independent experiments (*n* = 5) performed in triplicate. ^a^ *p* < 0.05 and ^b^ *p* < 0.01 compared to the data from untreated control group (control); ^c^ *p* < 0.05 and ^d^ *p* < 0.01 compared to the data from cells exposed to 6-OHDA with no treatment.

6-OHDA could enable the protein expression of Apaf-1 in SH-SY5Y cells to be 4.2-fold higher than the controls for untreated vehicles (Figure 4A). In cell treatment with HBA (120 μmol/L) after receiving 6-OHDA induction, the protein expression of Apaf-1 was reduced nearly by 46% (Figure 4A). The 6-OHDA induction expression of Apaf-1 was reduced to a greater extent when the cell was pretreated with NALC (1 mmol/L) or SP600125 (10 μmol/L) (reduced by 42.2% and 43.8%, respectively; Figure 4A).

The expression levels of both cleaved caspase-9 and -3 detected in the 6-OHDA induction cells were, respectively, 3.9- and 4.1-fold higher than the non-6-OHDA stimulation cells (Figure 4A). A significant increase in the protein expression of cleaved caspase-9 and -3 caused by 6-OHDA in the HBA (120 μmol/L) pre-treated group was less than 52.9% and 46.5% of the values for 6-OHDA induction, respectively (Figure 4A). Pretreatment cells with NALC (1 mmol/L) or SP600125 (10 μmol/L) following receiving 6-OHDA induction reduced the expression of cleaved caspase-9 by 39.6% and 51.6%, respectively (Figure 4A). High levels of cleaved caspase-3 in 6-OHDA-stimulated SH-SY5Y cells were reduced to 54.9% and 40.4% in cells that were pretreated with NALC (1 mmol/L) or SP600125 (10 μmol/L), respectively (Figure 4A).

6-OHDA made the cleaved PARP protein level in SH-SY5Y cells 3.5-fold higher than in the vehicle control group (Figure 4A). Pretreatment of SH-SY5Y cells with HBA (120 μmol/L) decreased the higher cleaved PARP protein levels caused by 6-OHDA at 44.1% (Figure 4A). NALC (1 mmol/L) or SP600125 (10 μmol/L) pretreatment reduced the effect of 6-OHDA on the expression of cleaved PARP protein in a 39.5% and 49.8% reduction, respectively (Figure 4A).

6-OHDA upregulated caspase-9 activity to 2.9-fold of the vehicle-treated counterparts, which lowered to 1.6-, 1.8-, and 1.5-fold in SH-SY5Y cells pretreated with HBA (120 μmol/L), NALC (1 mmol/L), or SP600125 (10 μmol/L), respectively (Figure 4B). The 2.7-fold higher caspase-3 activity in 6-OHDA-cultured SH-SY5Y cells was decreased (43.7, 40.6, and 45.7% reduction, respectively) by treatment with HBA (120 μmol/L), NALC (1 mmol/L), or SP600125 (10 μmol/L) when compared to those of the vehicle-treated counterparts (Figure 4B). The PARP activity in SH-SY5Y cells exposed to 6-OHDA was increased to 3.1-fold of that in the control group; HBA (120 μmol/L), NALC (1 mmol/L), and SP600125 (10 μmol/L) decreased the increased activity of PARP in cells exposed to 6-OHDA to 58.5, 59.3, and 56.7%, respectively (Figure 4B).

### 3.5. HBA Reduces Activation of the JNK/c-Jun Signal Induced by 6-OHDA

**Figure 5:**
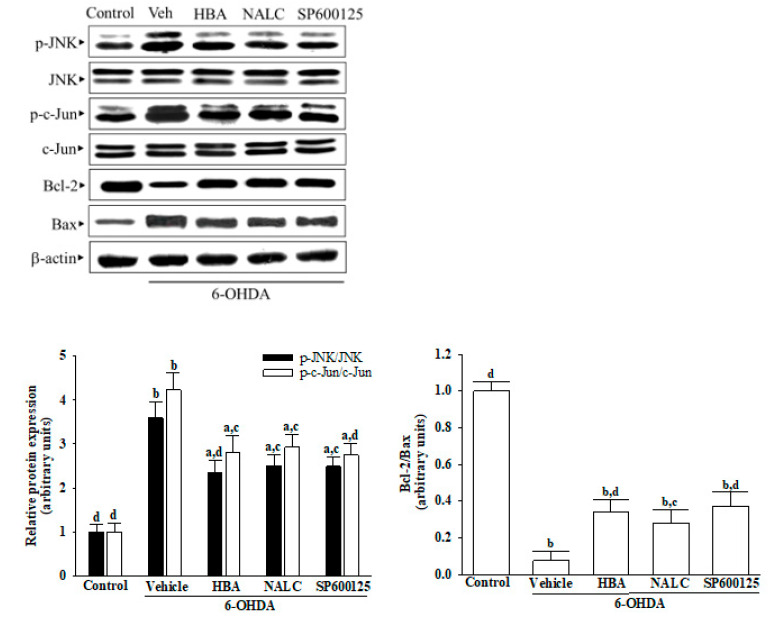
HBA reduces activation of the JNK/c-Jun signal induced by 6-OHDA. SH-SY5Y cells were pretreated with HBA (120 µmol/L), NALC (1 mmol/L), or SP600125 (10 µmol/L) for 1 h and then exposed to 6-OHDA (100 μmol/L) for another 24 h. Photographs representing Western transfer analysis for p-JNK, JNK, p-c-Jun, c-Jun, Bax, and Bcl-2. The ratio between phosphoprotein and total protein was calculated for JNK (p-JNK/JNK) and c-Jun (p-c-Jun/c-Jun). The ratio between the relative intensities of Bcl-2 and Bax (Bcl-2/Bax) has been indicated. The results are shown as the mean ± SD of five independent experiments (*n* = 5) performed in triplicate. ^a^ *p* < 0.05 and ^b^ *p* < 0.01 compared to the data from untreated control group (control); ^c^ *p* < 0.05 and ^d^ *p* < 0.01 compared to the data from cells exposed to 6-OHDA with no treatment.

The p-JNK/JNK and p-c-Jun/c-Jun ratios were 3.5- and 4.2-fold controls for untreated vehicles in the 6-OHDA-cultured cells, respectively (Figure 5). Pretreatment cells with HBA (120 μmol/L) declined the 6-OHDA-induced higher ratios of both p-pJNK/pJNK and p-c-Jun/c-Jun by 34.5 and 33.3%, separately; similar results were obtained when pre-treated with 1 mmol/L NALC (30.1% reduction in pJNK/pJNK and 30.7% reduction in p-c-Jun/c-Jun) or 10 μmol/L SP600125 (31.6% reduction in pJNK/pJNK and 34.9% reduction in p-c-Jun/c-Jun; Figure 5).

Immunoblotting results showed that 6-OHDA decreased the Bcl-2/Bax ratio in SH-SY5Y cells, which was approximately 7.5% of the normal group (Figure 5). The downregulated Bcl-2/Bax ratio by 6-OHDA appeared to 4.5-, 3.7-, and 4.9-fold elevation, respectively, in HBA (120 μmol/L), NALC (1 mmol/L), or SP600125 (10 μmol/L) pretreatment group (Figure 5).

## 4. Discussion

SH-SY5Y cells challenged with 6-OHDA have been extensively used as an in vitro model for PD; although the 6-OHDA model does not cover all symptoms of PD, it reproduces the main cellular processes involved in PD [19]. The neurotoxin 6-OHDA causes neuronal cell death through multiple pathways [19]. One of them, 6-OHDA, may accumulate within the cytosol, precede the formation of ROS and cause oxidative stress, and after that will cause neuronal death by apoptosis [19]. We first sought to evaluate cell viability to determine whether HBA, which rescues the cells from the neurotoxicity induced by 6-OHDA, was associated with fighting oxidative stress. Our findings demonstrated that HBA pretreatment significantly inhibited 6-OHDA-induced decreases in SH-SY5Y cell viability in addition to reversing the increase in ROS levels induced by 6-OHDA, which is consistent with earlier results that HBA had therapeutic effects on cells exposed to oxidative stress [17].

The antioxidant defense system removes cellular damage induced by free radicals during oxidative stress [27]. The commonly studied antioxidant enzymes SOD, CAT, and GSH-Px have protected against the deleterious effects of various oxidative stress paradigms [27]. The results showed that 6-OHDA-induced cytotoxicity in SH-SY5Y cells was accompanied by an evident decrease in the activities of SOD, CAT, and GSH-Px, confirming that 6-OHDA impaired the initial anti-oxidative defense systems, which may lead to its provoking of oxidative stress [19]. Thus, the antioxidant enzyme status of HBA pretreated SH-SY5Y cells was further evaluated. There was a significant increase in SOD in all the HBA-pretreated cells, proving that there was a direct activation of SOD by HBA to catalyze the superoxide anions produced by 6-OHDA. The increment in CAT activity observed in 6-OHDA-induced SH-SY5Y cells receiving HBA pretreatment demonstrated that HBA may activate the CAT enzyme, which catalyzed the toxic hydrogen peroxide to water and oxygen molecules. Our results also showed that HBA pretreatment tends to elevate GSH-Px in 6-OHDA-induced SH-SY5Y cells; that HBA helps GSH-Px reduce hydrogen peroxide and organic hydroperoxides to water or corresponding alcohols using GSH as an electron donor could thus be considerable. The effects of HBA on the inhibition of ROS overproduction and the increase in the expression of several endogenous antioxidants were similar to the action of NALC, an antioxidant and a ROS scavenger [28]. HBA efficiently inhibits the overproduction of ROS and enhances the activities of the first-line antioxidant defense, thus protecting the neuronal cell from PD-related neuronal injury induced by 6-OHDA. Although it has been considered that antioxidants have massive potential for preventing and delaying the development and progression of neurodegenerative diseases, treatment with supplementation of antioxidants appears insufficient if higher levels of ROS activate cell death processes [29]. Our results indicate a potential neuroprotective role of HBA due to its merit in reducing oxidative stress and providing enhanced antioxidant neuroprotection, while the needs remains to explore novel mechanisms further.

Once 6-OHDA is inside the mitochondria, it damages mitochondrial respiratory enzymes, interfering with mitochondrial respiration and reducing the potential of the mitochondrial membrane [30]. Loss of mitochondrial membrane potential results in reduced production of ATP and increases mitochondrial permeability [31]. The damage to the mitochondrial membrane is also closely associated with the Bcl-2 family of proteins, in which Bax can specially regulate the permeability of the mitochondrial extracorporeal membrane, causing increased release of cytochrome c from the mitochondria; Bcl-2 inhibits caspase activation and binds to Bax and other pro-apoptotic proteins [32]. The Bcl-2/Bax ratio is considered to be a better predictor of apoptosis than that of Bcl-2 or Bax on its own [33]. Once released from the mitochondria, cytochrome c interacts with Apaf-1 and, in concert with the apoptotic initiator caspase-9, forms the apoptosome that cleaves several downstream effector caspases, such as caspase-3, leading to DNA cleavage [34]. Caspase-3, when activated, can also cleave PARP-1, thus eliminating DNA repair to promote apoptosis [35]. As such, it is critical to the development of targeted mitochondrial therapies that have the potential to restore mitochondrial function, promote the survival of neuronal cells, and prevent neurodegeneration [5]. When SH-SY5Y cells were exposed to 6-OHDA, they exhibited a mitochondrial-mediated apoptotic phenomenon, including mitochondrial membrane potential loss, ATP depletion, cytochrome c release enhancement, caspase-9 and -3 activity increment, PARP cleavage promotion, as well as Bcl-2/Bax ratio up-regulation, which parallel with marked DNA fragmentation. All the above events were lessened under cells receiving HBA pretreatment, conferred that this compound exerted neuroprotective effects on SH-SY5Y cells against mitochondrial cytochrome c-activated caspases cascades induced by 6-OHDA, thus reducing apoptosis and recovery of cell viability. Thus, HBA candidates could be seen as capable of targeting mitochondrial defects to improve mitochondrial function and reduce neural death.

Neurotoxics and oxidative stress have been shown to activate JNKs, which then participate in a variety of cell functions, including apoptosis control, and can contribute to PD [6]. This unique feature makes JNK a promising target for extending pharmacological intervention [8]. Activated JNK rapidly induces the downstream target AP-1 transcription factor c-Jun, stimulates the expression of pro-apoptotic genes, and reduces the expression of pro-survival genes [36]. JNK mediates apoptosis not only through its effects on gene transcription but also through transcription-independent mechanisms that are involved in the intrinsic mode of cell death [36]. Inhibitors that block the association between JNK and mitochondria can be useful neuroprotectors in the treatment of PD [37]. JNK inhibition with a pan-JNK inhibitor SP600125 reduced mitochondrial dysfunction and blocked the intrinsic mitochondrial pathway to apoptosis in 6-OHDA-induced SH-SY5Y cells, with this reduced cell death programmed to maintain cell survival; HBA was just as effective as SP600125. These results support the direct link between the JNK pathway and mitochondrial apoptosis [4]. HBA protected neurons against deficiencies induced by 6-OHDA through extenuation of JNK/Jun/caspase-3 signaling pathway, which could be considerable. Unlike JNK1 and JNK2 that are expressed throughout the body, JNK3 is primarily expressed in the brain; therefore, JNK3 has been considered a potential therapeutic target for neurodegenerative diseases [38]. To determine whether HBA is different from the pan-JNK inhibitor but characterized by isoform selectivity at JNK3, additional explanations are necessary.

ROS and JNK are highly interlinked [39]. The same effect as HBA treatment with anti-oxidant NAC reduced intracellular ROS levels and was also associated with reduced activation of JNK and caspase-3 in response to exposure to 6-OHDA. However, protective effect of a phenol compound resveratrol attenuates the nigrostriatal pathway injury-induced neuronal apoptosis and inflammation through activation of JNK signaling has been documented [40]. Therefore, the neuroprotective effect of HBA arises from being an anti-oxidative agent to lower intracellular ROS levels, indirectly inactivating the JNK pathway, or being a primary blocker target to JNK needs more extensive study to clarify.

The administration of medications to cross the BBB remains a challenge in treating neurological diseases [41]. HBA can be absorbed well through passive diffusion into the intestine, and the absorption rates in the various intestinal segments do not show any regioselectivity [42]. In addition, there is evidence that HBA crosses the BBB using in vivo and in vitro models [43]. It showed that 32.91% of HBA could be used in the in vitro BBB model composed of cerebral endothelial cells after 240 min of dosing [44]. The pharmacokinetic parameters of the microdialysis-evaluated in vivo BBB permeability test in rats showed that HBA could go through BBB and reach its peak concentration at 40 min in blood and brain tissue [43]. HBA is bioavailable because it can be absorbed well and pass through the BBB, making it an ideal therapeutic candidate for PD and other neurological diseases.

Despite the promising potential of the HBA, our study has certain limitations. The main objective of our study was to evaluate the neuroprotective effect of HBA in an in vitro PD model; moreover, in vivo validation in animal models was necessary for future work. Benzyl alcohol may cause allergic reactions, but no serious adverse effects of benzyl alcohol have been reported in studies on chronically exposed animals in rats and mice [44]. Considering PD is a chronic disease that requires long-term therapy, as a result, further investigation into a broad safety profile of HBA becomes a critical issue with such a long dosing period to treat PD. In addition, carboxylations are generally selected to react with hydroxyl compounds to produce ester derivatives with enhanced biological activity [45]. It has been shown that 3-furancarboxylic acid diester from HBA is the most active sedative-hypnotic agent among HBA and its derivatives [45]. Thus, structural change of HBA through esterification could be an effective method for its application in modifying the function of the central nervous system [45]. In parallel with the above, the bioactive effects of HBA among its derivatives on protection against progressive neurodegenerative diseases warrant further assessment.

In conclusion, the protective effects of HBA against 6-OHDA-induced neurotoxicity were mediated through the induction of antioxidant enzymes to reduce oxidative stress conditions, resulting in mitochondrial protection and inhibiting cell apoptosis. The mechanisms of HBA to prevent apoptotic death in neuronal cells may also be associated with inhibiting the ROS-dependent JNK/Jun/caspase-3 signaling pathway. HBA should have therapeutic potential for the prevention and management of PD.

## Data Availability

All the data needed to evaluate the conclusions in the paper are present in the paper. Additional data related to this paper may be requested from the authors.
